# Marker-assisted introgression of wild chromosome segments conferring resistance to fungal foliar diseases into peanut (*Arachis hypogaea* L.)

**DOI:** 10.3389/fpls.2023.1139361

**Published:** 2023-03-17

**Authors:** Márcio de Carvalho Moretzsohn, João Francisco dos Santos, Andrea Rocha Almeida Moraes, Adriana Regina Custódio, Marcos Doniseti Michelotto, Namrata Maharjan, Soraya Cristina de Macedo Leal-Bertioli, Ignácio José Godoy, David John Bertioli

**Affiliations:** ^1^ Plant Genetics Laboratory, Embrapa Genetic Resources and Biotechnology, Brasília, DF, Brazil; ^2^ Grain and Fiber Center, Agronomic Institute of Campinas (IAC), Campinas, SP, Brazil; ^3^ Grain and Fiber Center, Agronomic Institute of Campinas (IAC), Pindorama, SP, Brazil; ^4^ Institute of Plant Breeding, Genetics and Genomics, University of Georgia, Athens, GA, United States; ^5^ Department of Plant Pathology, University of Georgia, Athens, GA, United States; ^6^ Department of Crop and Soil Science, University of Georgia, Athens, GA, United States

**Keywords:** *Arachis stenosperma*, *A. magna*, induced allotetraploid, late leaf spot, rust, molecular breeding

## Abstract

**Introduction:**

Fungal foliar diseases can severely affect the productivity of the peanut crop worldwide. Late leaf spot is the most frequent disease and a major problem of the crop in Brazil and many other tropical countries. Only partial resistance to fungal diseases has been found in cultivated peanut, but high resistances have been described on the secondary gene pool.

**Methods:**

To overcome the known compatibility barriers for the use of wild species in peanut breeding programs, we used an induced allotetraploid (*Arachis stenosperma* × *A. magna*)^4x^, as a donor parent, in a successive backcrossing scheme with the high-yielding Brazilian cultivar IAC OL 4. We used microsatellite markers associated with late leaf spot and rust resistance for foreground selection and high-throughput SNP genotyping for background selection.

**Results:**

With these tools, we developed agronomically adapted lines with high cultivated genome recovery, high-yield potential, and wild chromosome segments from both *A. stenosperma* and *A. magna* conferring high resistance to late leaf spot and rust. These segments include the four previously identified as having QTLs (quantitative trait loci) for resistance to both diseases, which could be confirmed here, and at least four additional QTLs identified by using mapping populations on four generations.

**Discussion:**

The introgression germplasm developed here will extend the useful genetic diversity of the primary gene pool by providing novel wild resistance genes against these two destructive peanut diseases.

## Introduction

Cultivated peanut (*Arachis hypogaea* L.) is an important oilseed crop, grown throughout the tropics and sub-tropics. It is also widely used for human and animal consumption being a valuable source of dietary protein. Globally, 53.9 million tons of unshelled peanut were produced in 2021 in 32.7 million ha (FAOSTAT – Food and Agriculture Organization of the United Nations, 2022). Brazil only produced approximately 1.4% of this total; however, the crop is expanding in the country. In the last decade, the national production and the area planted increased 164% and 96%, respectively, reaching 746,700 tons of unshelled peanut harvested in 200,100 ha in the 2021/2022 season (CONAB - Companhia Nacional de Abastecimento, 2023). Peanut production in Brazil is highly concentrated in the state of São Paulo. In 2021/2022, São Paulo produced 692,700 tons of unshelled peanuts (approximately 90% of the Brazilian peanut production), of which more than 50% were shelled and processed for export (CONAB - Companhia Nacional de Abastecimento, 2023). Breeding programs are active in the country and releasing new cultivars to attend to this market. However, it is necessary to prepare this chain of production to be more competitive, especially through reducing costs of production, such as reducing dependence on fungicides.

The genus *Arachis* is native to South America. It contains 83 described species, assembled into nine taxonomic sections according to their morphology, geographical distribution, and cross-compatibility relationships (Krapovickas and Gregory, 1994; Valls and Simpson, 2005; Valls et al., 2013; Valls and Simpson, 2017; Seijo et al., 2021). Cultivated peanut belongs to section *Arachis*, which also includes 32 closely related wild species. Of these, 28 are diploid with *x* = 10 (2*n*=20), three species are diploid with *x* = 9 (2*n*=18), and *A. hypogaea* and *A. monticola* Krapov. & Rigoni are allotetraploids (2*n*=4*x*=40) with a genome formula AABB (Lavia et al., 2009; Stalker, 2017 and references therein; Seijo et al., 2021). Six genome types, A, B, D, F, K, and G, have been described for the diploid species in section *Arachis*, differing on the chromosome morphology, distribution patterns of heterochromatic bands and rDNA loci, and cross-compatibility (Smartt et al., 1978; Stalker, 1991; Fernandez and Krapovickas, 1994; Robledo and Seijo, 2010; Silvestri et al., 2015).

*Arachis hypogaea* has a narrow genetic base and is susceptible to various biotic stresses. Among them, fungal foliar diseases, especially late leaf spot (LLS) [*Nothopassalora personata* syn. *Cercosporidium personatum* (Berk. & M.A. Curtis) S.A. Khan & M. Kamal], early leaf spot (*Passalora arachidicola* syn. *Cercospora arachidicola* S. Hori), and rust (*Puccinia arachidis* Speg.), are widespread in most of the producing countries and severely affect productivity. Reductions of up to 70% in peanut yield caused by leaf spots have been described (Backman and Crawford, 1984; Singh et al., 2011; Coutinho and Suassuna, 2014). LLS is the most frequent disease and a major problem of the crop in Brazil. The disease starts at 45–50 days after germination and tends to progress until the end of the crop cycle. Fungus sporulation occurs on the abaxial surface of the leaves and disease symptoms appear approximately 10 days after infection (Shokes and Culbreath, 1997). The main symptom is the presence of round-shaped black lesions on the leaves, which reduces the foliar area and induces defoliation. This causes a reduction in photosynthesis and consequently decreases peanut productivity. Rust is a sporadic disease, but can be highly destructive. Initial symptoms are small yellow round lesions on both surfaces of the leaves, and subsequently, these lesions turn to reddish brown. Unlike LLS, rust-infected leaves tend to remain attached to the plant, which favors the pathogen multiplication and rapid spread of the disease (Moraes and Godoy, 1997). The main damage of rust is the reduction of foliar area available for photosynthesis. To control both diseases, multiple fungicide sprays are needed throughout the growing season, usually at intervals of up to 15 days, depending on weather conditions. Although several fungicides are available, their application significantly increases crop management costs and the risks of soil and environment contamination. Resistant varieties are considered the most efficient way to control these diseases. Few, and only moderate, resistance sources to fungal diseases have been found in *A. hypogaea*, but high resistance has been described for many *Arachis* wild species (reviewed by Stalker, 2017). Therefore, there is a growing need to improve peanut resistance to fungal foliar diseases by diversifying its genetic variability utilizing crop wild relatives.

Since most *Arachis* wild species are diploid and *A. hypogaea* is an allotetraploid, the introgression of useful wild genes into peanut is not a trivial task. To overcome this, interspecific induced allotetraploid plants have been developed (Burow et al., 2001; Fávero et al., 2006; Kumari et al., 2014; Leal-Bertioli et al., 2015b; Leal-Bertioli et al., 2017; Gao et al., 2021; Bertioli et al., 2021b). In this so-called tetraploid route (Simpson, 1991), species with A and B genomes are intercrossed and the resulting sterile hybrid (AB) is treated with colchicine to duplicate the chromosomes and restore fertility (AABB). The allotetraploid thus obtained can be crossed with *A. hypogaea* to produce fertile hybrids.

Specially for introgression of wild genes, marker-assisted backcrossing (MABC) is essential, as it enables a rapid recovery of the recurrent parent genome, the pyramiding of multiple useful genes into the same genotype, and considerable time saving (Frisch and Melchinger, 2005; Bharadwaj et al., 2022; Kim et al., 2022). Nevertheless, very few genomic segments of *Arachis* wild species containing genes of interest have been well-defined to date. Regarding resistance to biotic stresses, a few segments from the species *A. cardenasii* Krapov. & W.C.Greg. (A genome) with genes for resistance to root-knot nematode (RKN) (Chu et al., 2007), leaf spots, rust, and web blotch (Kolekar et al., 2016; Pandey et al., 2017b; Ahmad et al., 2020; Lamon et al., 2020; Bertioli et al., 2021a) were identified. By using marker-assisted selection, segments conferring RKN resistance were introgressed into released peanut cultivars (Chu et al., 2011; Simpson et al., 2013; Clevenger et al., 2017b). In addition, both by anonymous phenotypic and by marker selection, *A. cardenasii*-containing segments that confer resistance to leaf spots and rust have been introgressed into peanut in breeding programs worldwide (Varshney et al., 2014; Shasidhar et al., 2020; Godoy et al., 2022; Holbrook et al., 2022). The very large-scale anonymous contribution of *A. cardenasii* to the peanut crop was recently shown with high-throughput genotyping; 251 peanut lines and cultivars in 30 countries were found to have genetics from this wild species; in almost all cases, the breeders involved were unaware of the wild genetics (Bertioli et al., 2021a).

In previous studies, we identified wild genomic segments conferring resistance to LLS and to RKN in *A. stenosperma* Krapov. & W.C.Greg. (Leal-Bertioli et al., 2009, Leal-Bertioli et al., 2016; Ballén-Taborda et al., 2019), and a robust QTL (quantitative trait locus) for resistance to rust in *A. magna* Krapov., W.C.Greg. & C.E.Simpson (Leal-Bertioli et al., 2015a). *Arachis stenosperma* (A genome 2*n*=2*x*=20) is an annual plant, belongs to section *Arachis*, and is endemic to Brazil (Krapovickas and Gregory, 1994), where it was cultivated for food by native people (Simpson et al., 2001). *Arachis magna* (B genome, 2*n*=2*x*=20) is also annual, belongs to section *Arachis*, and has been collected in Brazil and Bolivia (Krapovickas and Gregory, 1994; Custódio et al., 2013). An induced allotetraploid (2*n*=4*x*=40) was previously developed using these species and found to be resistant to both rust and LLS (Fávero et al., 2015a; Michelotto et al., 2016; and unpublished data). In the present study, this induced allotetraploid was used as the donor parent in successive backcrossings with an elite Brazilian cultivar for the incorporation of wild resistance QTLs into cultivated peanut. We used molecular markers for the selection of plants containing the resistance QTLs with a lower proportion of wild chromosome segments for a faster recovery of the recurrent *A. hypogaea* genome. The plants obtained were assayed in the field each year, supporting the hypothesis that the selected chromosome regions of *A. stenosperma* and *A. magna* conferred resistance to otherwise susceptible plants. Furthermore, we identified additional QTLs controlling LLS and rust.

## Material and methods

### Plant material

The recurrent parent ‘IAC OL 4’ (*A. hypogaea* subsp. *hypogaea* var. *hypogaea*) is a commercial peanut cultivar developed by the Instituto Agronômico (IAC), Campinas, Brazil (Godoy et al., 2014). It is a high oleic runner cultivar widely grown in São Paulo state, highly yielding, but very susceptible to foliar diseases. The wild donor parent was the induced allotetraploid (*A. magna* K 30097 × *A. stenosperma* V 15076)^4x^ (Fávero et al., 2015b), hereafter called MagSten. This induced allotetraploid and its diploid parents have high resistance to foliar fungal diseases (Michelotto et al., 2015, Michelotto et al., 2016). The diploid species were obtained from the *Arachis* Germplasm Collection, maintained at Embrapa Genetic Resources and Biotechnology (Brasília-DF, Brazil).

### Marker-assisted backcrossing scheme

**Figure 1:**
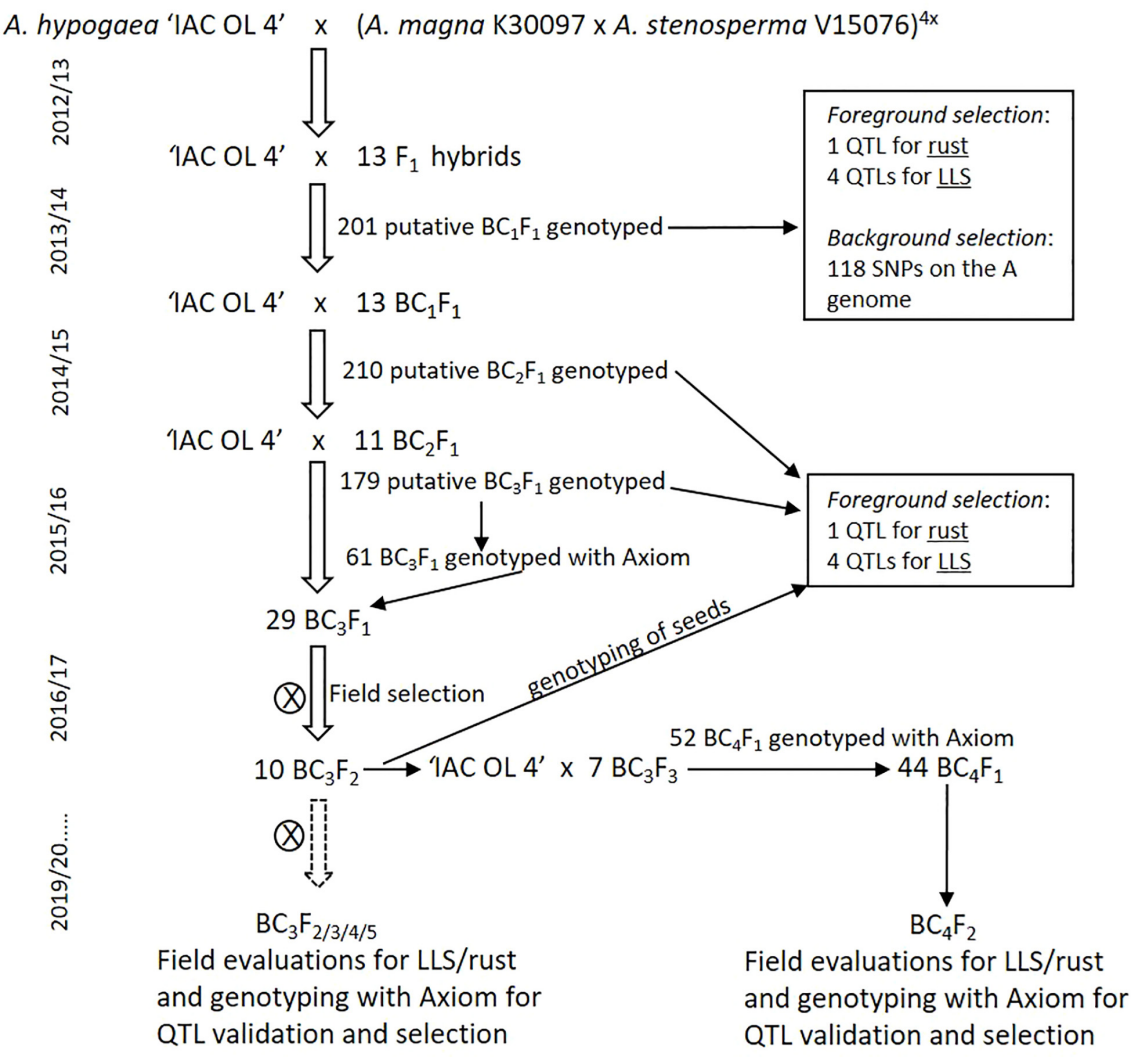
Marker-assisted backcrossing schedule for introgressing chromosome segments from *A. magna* K 30097 and *A. stenosperma* V 15076 into the peanut cultivar IAC OL 4.

The first round of crosses was made in 2012 using ‘IAC OL 4’ as the female and MagSten as the male parent (**Figure 1**). True F_1_ hybrids were identified by microsatellite markers, grown in a greenhouse, and used as male parents on the first backcross with ‘IAC OL 4’. Using ‘IAC OL 4’ as a recurrent parent, generations were advanced to BC_4_ from 2013 to 2017/2018. On each backcross cycle, progenies were selected using microsatellite markers linked to known desirable QTLs, essentially as described by Moretzsohn et al. (2013). These included a QTL for rust resistance from *A. magna* K 30097 (on chromosome B08; Leal-Bertioli et al., 2015a) and four QTLs for LLS resistance, located on three chromosomes (A02, A06, and two segments on the middle of A04; Leal-Bertioli et al., 2009) (**Supplementary file 1**). These QTLs were identified in a different *A. stenosperma* accession (V 10309), but tentatively used on the accession V 15076 also highly resistant to fungal foliar diseases (Michelotto et al., 2015), and the one incorporated into MagSten. Most of the backcrossings and selfings were also accompanied by different SNP genotyping methods for monitoring the introgressed wild segments and for faster recovery of *A. hypogaea* genetic background. BC_1_F_1_ plants were genotyped with SNP markers dispersed through the 10 A-genome chromosomes using a 384 Illumina BeadXpress array (**Supplementary file 1**). The BC_2_ generation was not submitted to background selection. BC_3_F_1_ plants were genotyped with the first version of the Thermofisher SNP array (Axiom_*Arachis* 58k array) that assayed 58,233 SNP markers (Clevenger et al., 2017a; Pandey et al., 2017a), while BC_3_F_2_ to BC_3_F_5_, BC_4_F_1_, and BC_4_F_2_ plants were genotyped with the improved array version (Axiom_*Arachis* v.02) that assays 48,000 SNP markers (Korani et al., 2019). These populations were evaluated for resistance to fungal foliar diseases, pod weight, number of seeds per pod, and pod shape (cultivated, mixed or wild type) in field assays. Data on genotypic and field evaluation were used for the selection of progenies to be advanced to the next generation.

### Field evaluations

Five field trials were conducted in the APTA (Agência Paulista de Tecnologia dos Agronegócios) experimental station in Pindorama, São Paulo State, located in the most important peanut-producing state of Brazil, using generations BC_3_F_2_, BC_3_F_3_, BC_3_F_4_, BC_3_F_5_, and BC_4_F_2_. Thirty seeds per selected family were planted in 15-m-long single-randomized row plots, using ‘IAC OL 3’ as susceptible control, and ‘IAC Caiapó’ and ‘IAC Sempre Verde’ as resistant checks. MagSten was also included in some of the trials. Field management followed standard technical recommendations for the crop, except for disease control as no fungicide was sprayed. At approximately 120 days after sowing, each plant was evaluated for LLS incidence using the grade scale ranging from “1” (no spots or defoliation) to “9” (many spots on the remaining leaves and a severe defoliation), according to Subrahmanyam et al. (1995), but including the 0.5 scores for intermediate aspects between adjacent integer scores. Rust is a sporadic disease and the incidence was low on four of the five assays. Therefore, it was evaluated using “1” for the presence and “0” for absence of rust lesions in leaves. On the BC_4_F_2_ field assay, rust incidence was moderate and the plants were evaluated using the 1–9 grade scale.

### Mapping QTLs for rust and LLS resistance

Phenotypic and genotypic data obtained here were used not only for MABC, but also for QTL mapping. From BC_1_ to BC_4_, the progenies were submitted to a stringent foreground selection. Therefore, most selected plants had different combinations of the four wild chromosome segments of interest, in A02, A06, B08, and A04 (with two QTLs located closely to each other), some wild genomic segments not marker-selected (“linkage drag”) and a cultivated background on most of the chromosomes. Those populations are not ideal for QTL mapping, as the plants had been marker-selected and some wild chromosome segments had been lost on all the analyzed plants. However, they were used with this aim, as an additional test to validate the association of previous identified QTLs with disease resistances, since they were present in plants of the four populations. Loci homozygous for the cultivated alleles were discarded and the remaining loci were submitted to QTL analysis using the nonparametric interval mapping based on the Kruskal–Wallis test statistic, performed in R/qtl (Broman et al., 2003). The *scanone* function and the argument model=“np” were used for LLS resistance, and the argument model=“binary” was used for rust resistance. We also used the model=“np” for rust resistance in BC_4_F_2_, since the plants were evaluated using the 1–9 grade scale. To increase the reliability, GWAS (genome-wide association study) was conducted using the GAPIT and FarmCPU model in R. Kinship matrix was generated using whole-genome SNPs from Affymetrix data in TASSEL. Significance threshold was calculated based on FDR value at 0.05. The BC_3_F_3_ field assay was not included on QTL analyses and GWAS due to the low number of plants assayed.

## Results

### Marker-assisted development of advanced lines

The induced allotetraploid MagSten was used as a donor parent in crosses with ‘IAC OL 4’ (**Figure 1**). Thirteen true F_1_ hybrids identified by microsatellite markers were crossed with ‘IAC OL 4’. A total of 201 putative BC_1_F_1_ seeds were obtained, planted in greenhouses, and genotyped with microsatellite markers linked to the five QTLs for resistance to LLS and rust. For monitoring the percentages of genome donor in each progeny, we also genotyped the 201 plants with 384 SNPs dispersed through the 10 A-genome chromosomes developed for an *A. duranensis* × *A. stenosperma* cross based on EST (Expressed Sequence Tags) data (**Supplementary file 1**). This was the highest coverage genotyping method available for *Arachis* at that time (2014). A total of 118 SNPs were informative. The proportion of wild genome ranged from 9.1% to 40.8%, with an average of 22.7% on the 201 analyzed plants. Seventeen self-pollinated plants were discarded. Thirteen BC_1_F_1_ plants were selected based on the presence of at least four of the five resistance QTLs and smaller percentage of donor (wild) A genome, ranging from 12.7% to 25.9% on the selected 13 plants. The 13 BC_1_F_1_ plants were used as male parents in backcrosses with ‘IAC OL 4’ to produce the BC_2_ generation. In total, 210 putative BC_2_F_1_ plants were genotyped with the microsatellite markers linked to the five QTLs. Twenty-six plants were discarded as self-pollinations and 11 were selected based on the presence of at least three of the five desired QTLs and used as male parents in backcrosses with ‘IAC OL 4’. One hundred seventy-nine putative BC_3_F_1_ plants were obtained and genotyped with the same microsatellite markers. Sixty-one true BC_3_F_1_ plants were genotyped using the Thermofisher Axiom *Arachis* v.01 (Clevenger et al., 2017a; Pandey et al., 2017a). Twenty-nine plants, with low genome donor (ranging from 2.3% to 12.2%), were chosen and selfed to produce BC_3_F_2_ seeds. BC_3_F_2_ plants were tested in the field, as described below, and 10 plants with LLS scores below “3”, good pod production (over 100 g/plant, not shown), and cultivated-like pod shape were selected. Slices of 30 seeds from each of the 10 plants were genotyped with microsatellite markers linked to the resistance QTLs, and seven plants (BC_3_F_4_) were backcrossed with ‘IAC OL 4’ to’ produce the BC_4_ generation (**Figure 1**). Fifty-two seeds were obtained, planted in greenhouses, and genotyped with the Axiom chip. The 44 true BC_4_F_1_ plants were selfed to produce BC_4_F_2_ seeds. Besides BC_3_F_2_, field assays were conducted on BC_3_F_3_, BC_3_F_4_, BC_3_F_5_, and BC_4_F_2_ plants. Foliar disease resistance, pod traits, and SNP genotyping data were used for the selection of plants to be advanced by selfing to the next generation.

### Field assays

Plants of five generations (BC_3_F_2_, BC_3_F_3_, BC_3_F_4_, BC_3_F_5_, and BC_4_F_2_) were assayed on the field for resistance to LLS and rust, pod shape, and pod production under disease pressure. ‘IAC Sempre Verde Caiapó’, and ‘IAC OL 3’, which are resistant, partly resistant, and susceptible to both diseases, respectively, were used as controls. All populations showed genetic variation for LLS resistance and pod weight (**Tables 1** and **2**).

**Table 1:** Number of plants evaluated (*n*), LLS score range, and means of plants with introgressions from *Arachis stenosperma* and *A. magna* of generations BC_3_F_2_ to BC_3_F_5_, and BC_4_F_2_.

Generation	*n*	LLS range	LLS mean (SD)	‘Sempre Verde’	‘Caiapó’	‘OL 3’
BC_3_F_2_	290	1.5–8.0	6.0 (1.5)^a B^	2.9 (0.2)^C^	6.3 (0.7)^B^	7.9 (0.4)^A^
BC_3_F_3_	72	2.0–7.5	4.4 (1.4)^b A^	1.6 (0.2)^B^	4.9 (0.8)^A^	–
BC_3_F_4_	474	2.5–9.0	6.3 (1.5)^a B^	3.1 (0.7)^C^	7.4 (0.3)^AB^	8.7 (0.4)^A^
BC_3_F_5_	141	1.0–6.0	2.5 (1.0)^c A^	2.0 (0.5)^A^	5.8 (0.9)^B^	–
BC_4_F_2_	258	1.0–7.5	4.0 (1.4)^b B^	1.4 (0.3)^C^	4.1 (1.2)^B^	7.0 (0.0)^A^

Means followed by the same uppercase letter in rows and the same lowercase letter in columns are not significantly different by the Tukey test at 5% probability, performed by the R package "agricolae" version 1.3.5 (Mendiburu, 2021).

Means (and standard deviation) of the checks ‘IAC Sempre Verde’, ‘IAC Caiapó’, and ‘IAC OL 3’ are also shown. ‘OL 3’ plants of generations BC3F3 and BC3F5 were not evaluated.

**Table 2:** Number of plants evaluated (*n*), pod weight range per plant, and mean (g) of generations BC_3_F_2_, BC_3_F_3_, BC_3_F_5_, and BC_4_F_2_ of selected plants with introgressions from *Arachis stenosperma* and *A. magna*.

Generation	*n*	Pod weight range	Pod weight mean (SD)	‘Sempre Verde’	‘Caiapó’	‘OL 3’
BC_3_F_2_	290	24.0–521.0	157.0 (78.2)^bc A^	207.5 (71.5)^A^	194.6 (68.8)^A^	139.1 (79.7)^B^
BC_3_F_3_	72	23.2–456.7	167.5 (105.8)^ab A^	344.3 (169.4)^A^	234.0 (71.7)^A^	–
BC_3_F_5_	141	30.1–417.2	185.4 (88.5)^a A^	377.1 (91.3)^B^	170.0 (73.3)^A^	–
BC_4_F_2_	258	11.6–410.2	142.1 (70.2)^c A^	215.6 (80.4)^A^	145.3 (69.0)^A^	194.4 (74.3)^A^

Means followed by the same uppercase letter in rows and the same lowercase letter in columns are not significantly different by the Tukey test at 5% probability, performed by the R package "agricolae" version 1.3.5 (Mendiburu, 2021).

Means (and standard deviation) of the checks ‘IAC Sempre Verde’, ‘IAC Caiapó’, and ‘IAC OL 3’ are also shown. Pod production of ‘OL 3’ in generations BC3F3 and BC3F5 was not evaluated.

A total of 290 individuals of 10 of the 12 selected BC_3_F_2_ families (two of them produced few seeds) were evaluated in 2017/2018. For comparison, 141 individuals of seven BC_3_F_2_ families that did not carry any, or only a few, of the four wild genomic segments of interest were also included. LLS scores ranged from 1.5 to 8.0, averaging 6.0 within the 10 selected families (**Table 1**) and ranged from 3.0 to 8.5 with an average of 7.2 within the seven check families. MagSten, ‘IAC Sempre Verde’, ‘IAC Caiapó’, and ‘IAC OL 3’ plants averaged 1.1, 2.9, 6.3, and 7.9, respectively. Rust lesions were detected in 102 out of the 290 plants from the selected families (35.2%) and on 97 out of the 141 plants of the check families (68.9%). For the controls, rust lesions were found on 24 of the 42 ‘IAC Caiapó’, 15 of the 16 ‘IAC Sempre Verde’, and all the 34 ‘OL 3’ plants. No symptoms were observed on the six MagSten plants. Population pod weight ranged from 24.0 g to 521.0 g, with an average of 157.0 g (**Table 2**), higher than the recurrent parent ‘OL 3’ n-significantly different from ‘IAC Sempre Verde’ and ‘IAC Caiapó’. Twenty-five plants were selected based on LLS scores (below 4.5), no symptoms of rust, the presence of most of the desired wild genomic segments, individual pod production (above 100 g per plant), and pods as similar as possible to the Runner shape. These same traits were used for the selection of plants on subsequent generations, except the LLS resistance score that varied on each trial.

Seeds of the 25 BC_3_F_2_ plants were planted in 2018/2019. A severe drought impaired plant growth and only 72 BC_3_F_3_ plants were evaluated for LLS resistance. Average LLS scores of the 72 plants ranged from 2.0 to 7.5, with an average of 4.4 (**Table 1**). ‘IAC Sempre Verde’ and ‘IAC Caiapó’ averaged 1.6 and 4.9, respectively. Incidence of rust was very scarce and observed on only four plants (5.6%). Despite the severe drought, pod production was relatively high, numerically comparable to the other trials: pod weight ranged from 23.2 g to 456.7 g, with an average of 167.5 g (**Table 2**).

Ten plants with LLS scores lower than 3.0 and the other desirable traits were selected and their seeds were planted in the field. LLS scores of the resulting 474 BC_3_F_4_ plants ranged from 2.5 to 9.0, averaging 6.3 (**Table 1**). MagSten, ‘IAC Sempre Verde’, ‘IAC Caiapó’, and ‘IAC OL 3’ plants averaged 1.0, 3.1, 7.4, and 8.7, respectively. Rust lesions were detected in 54.2% of the BC_3_F_4_ plants, in 17% of the ‘IAC Sempre Verde’ plants, and in all ‘IAC Caiapó’ and ‘IAC OL 3’ plants. No rust lesions were observed on the MagSten plants.

Forty BC_3_F_4_ plants were selected for having LLS scores lower than 4.5 and the other desirable traits (in this case, individual production was not weighed, but assessed visually). Seeds were planted, and 141 BC_3_F_5_ plants reached maturity and were evaluated. LLS scores ranged from 1.0 to 6.0, with an average of 2.5 and only six plants (4.3%) showed some few rust lesions (**Table 1**). LLS scores for ‘IAC Sempre Verde’ and ‘IAC Caiapó’ plants averaged 2.0 and 5.8, respectively. Pod weight per plant ranged from 30.1 g to 417.2 g, with an average of 185.4 g (**Table 2**). A total of 66 plants with LLS scores lower than 2.0, no rust lesions, with the desired wild segments, and good yield and pod/kernel traits were selected to be advanced. One of these plants was scored as 2.0 for LLS resistance, the same as ‘IAC Sempre Verde’, the highly resistant check, but it was more productive (417.2 g against 377.1 g). In addition, two plants were more resistant to LLS (scored as 1.0), with production comparable to ‘Sempre Verde’ (**Supplementary file 2**). None of these three plants had rust lesions, while two of the six ‘Sempre Verde’ showed some lesions.

**Figure 2:**
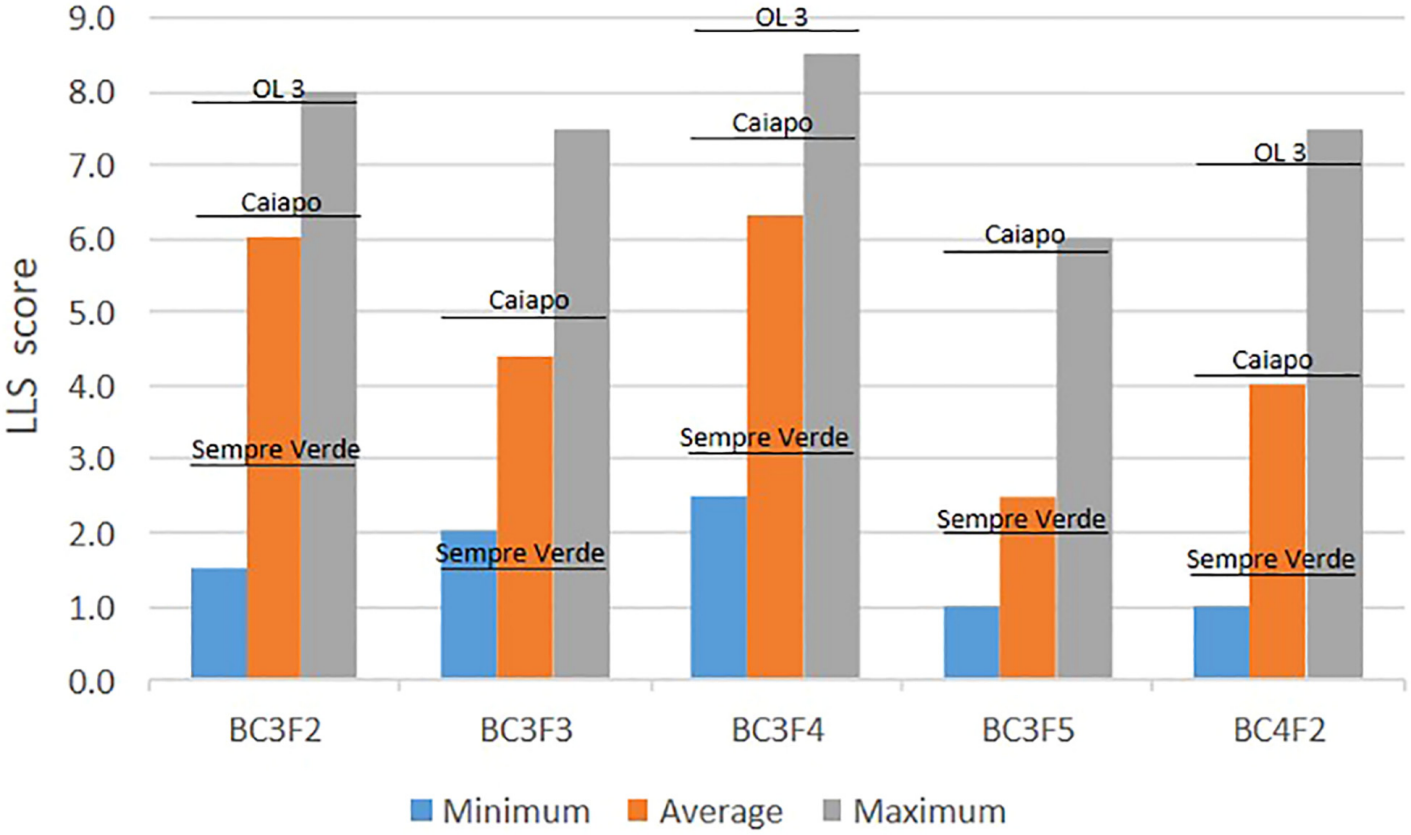
Minimum (blue), average (orange), and maximum (gray) LLS scores of 1,235 plants with introgressions from *Arachis stenosperma* and *A. magna* of generations BC_3_F_2_, BC_3_F_3_, BC_3_F_4_, BC_3_F_5_, and BC_4_F_2_, and average scores of the checks ‘IAC Sempre Verde’, ‘IAC Caiapó’, and ‘IAC OL’.

The BC_4_F_2_ generation was planted in the field in the 2019/2020 growing season for another round of genotypic and phenotypic selection for disease resistance, in a total of 258 plants. LLS scores ranged from 1.0 to 7.5, averaging 4.0. ‘IAC Sempre Verde’, ‘IAC Caiapó’, and ‘IAC OL 3’ plants averaged 1.4, 4.1, and 7.0, respectively. This season, rust incidence enabled us to score disease severity: Rust scores averaged 1.5, and varied from 1.0 to 5.0. ‘IAC Sempre Verde’, ‘IAC Caiapó’, and ‘IAC OL 3’ plants averaged 1.4, 1.5, and 2.0, respectively. Pod weight ranged from 11.6 g to 410.2 g, with an average of 142.1 g. With this evaluation and based on the presence of the wild segments of interest, 67 plants rating 1 to 3 and with the other desired traits were selected for future evaluation and selection. Two of these plants were as resistant to LLS as ‘IAC Sempre Verde’, and one plant yielded the same while the other produced considerably more than ‘Sempre Verde’ (311.8 g against 215.6 g) (**Supplementary file 2**). A summary of the results of these five field assays is shown in **Figure 2**.

### Mapping QTLs for LLS and rust resistance

A total of 1,204 plants of BC_3_F_2_, BC_3_F_3_, BC_3_F_4_, BC_3_F_5_, and BC_4_F_2_ generations were both field assayed and genotyped with the Thermofisher Axiom Array (**Supplementary file 2**). These data helped us on the selection of plants on each generation, but was also used for QTL mapping and to validate the association of previously identified QTLs with resistance against both diseases.

**Figure 3:**
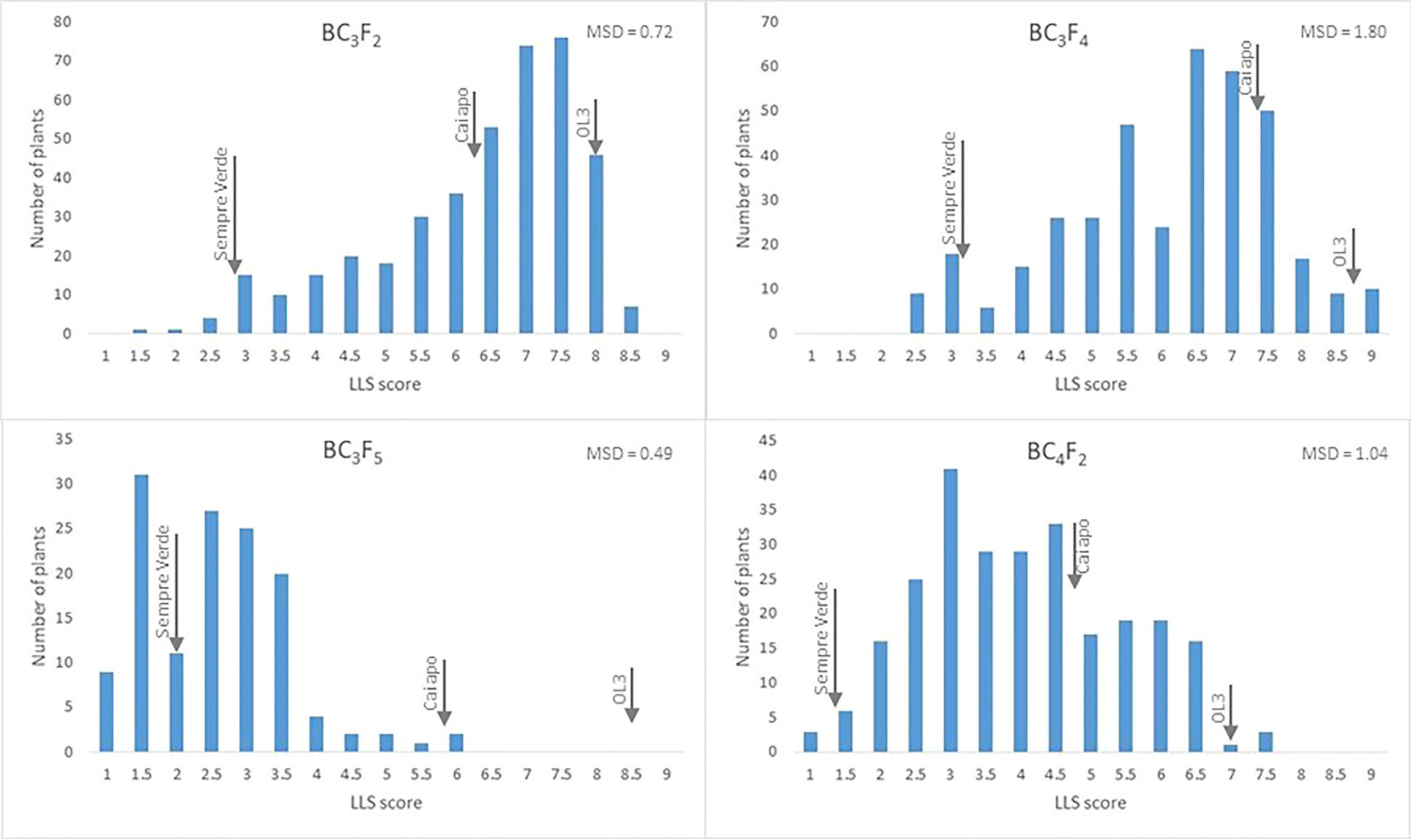
Frequency distribution of LLS resistance in four populations derived from 'IAC OL 4' × MagSten crosses. ‘IAC Sempre Verde’ (resistant), ‘IAC Caiapó’ (partly resistant), and ‘IAC OL 3’ (susceptible) were used as controls, and their average scores were indicated by arrows. MSD is the minimum significant difference estimated by the Tukey test at 5% probability.

The skewness, kurtosis, and normality test by *χ* ^2^, estimated with WinQTL Cartographer 2.5 (Wang et al., 2006), showed that the scores of LLS incidence on the four generations, as well as the rust resistance on BC_4_F_2_, were non-normally distributed (data not shown). We were unable to find any transformation of data that approximated the distributions to normality, and the nonparametric interval mapping was performed for QTL detection. The frequency distribution of plants according to their scores for LLS incidence showed bias toward susceptibility in the generations BC_3_F_2_ and BC_3_F_4_, and, in contrast, toward resistance on generations BC_3_F_5_ and BC_4_F_2_ (**Figure 3**). For rust severity (on BC_4_F_2_ only), the frequency distribution was strongly biased toward resistance, and 148 plants were evaluated as 1.0, and 87 as 2.0. Only 22 plants had scores ranging from 3.0 to 5.0. Pearson’s correlation between LLS scores and rust incidence was 0.624 for the 1,204 plants (not significant at 5% probability by the *t*-test). However, there was a clear tendency for plants more resistant to LLS to have fewer rust lesions (**Supplementary file 2**). The correlations between pod weight per plant and LLS scores and rust incidence were −0.177 and −0.106, respectively, both not significant at 5% probability by the *t*-test.

**Figure 4:**
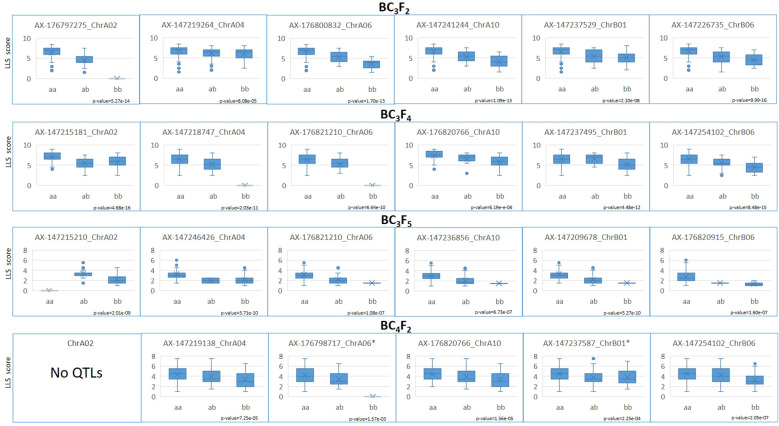
Boxplots showing the significant differences of LLS resistance scores between individuals homozygous for the cultivated (aa) and wild (bb) alleles, and heterozygous (ab) for the nearest loci of QTLs identified on chromosomes A02, A04, A06, A10, B01, and B06, on populations BC_3_F_2_, BC_3_F_4_, BC_3_F_5_, and BC_4_F_2_. Genotypes not observed are shown as x with a score of 0. No QTL was detected on the chromosome A02 on BC_4_F_2_.

**Figure 5:**
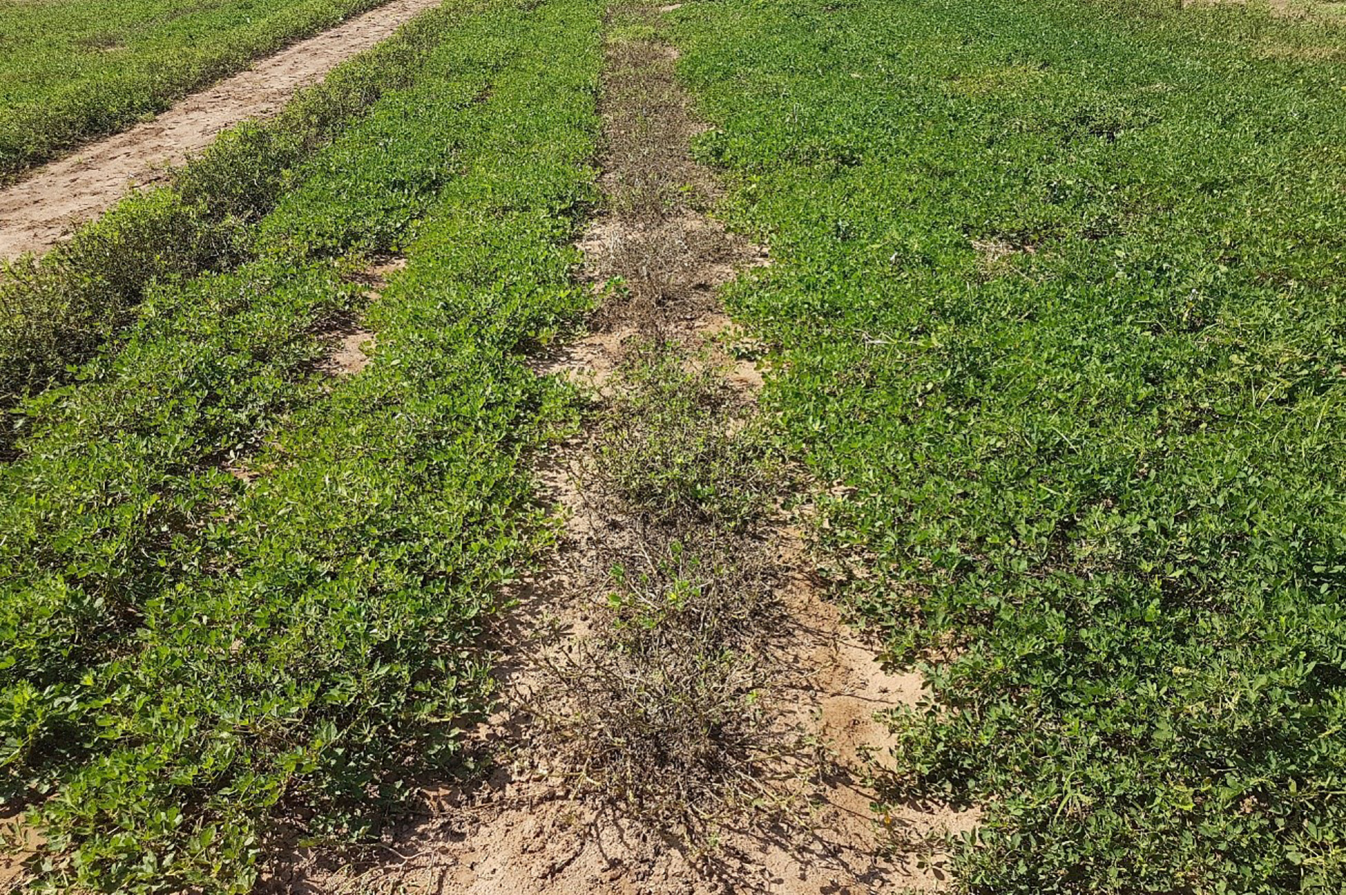
Field trial of BC_3_F_5_ plants descending from ‘IAC OL 4’ × (*A. magna* K30097 × *A. stenosperma* V15076)^4x^. Disease pressure was high and no fungicide sprays were applied. Field at the middle of the season. Mid row is the susceptible control ‘IAC OL 3’, highly affected by LLS, and left and right rows are BC_3_F_5_ plants with chromosome segments conferring disease resistance.

On the BC_3_F_2_ assay, with 406 genotyped plants, 1,133 loci were informative, since they were heterozygous or homozygous for the wild allele. These loci were scattered throughout the 20 chromosomes. Markers associated with LLS resistance were significantly (*p* < 0.01) mapped in all but four chromosomes (A05, A08, B04, and B10) using the nonparametric interval mapping (**Supplementary file 3**). The major QTLs, detected with higher LOD scores, were located on A02, B06, A10, A06, B01, and A04 in descending order of LOD scores (**Table 3**). Due to the small number of plants obtained, data from the BC_3_F_3_ population was not used for QTL mapping. For the BC_3_F_4_ plants, with 380 genotyped plants, 1,002 loci scattered through 18 chromosomes had wild alleles (all the 85 loci of chromosome A08 and the 66 loci of B02 informative in BC_3_F_2_ were homozygous for the cultivated allele). Loci linked to LLS resistance were significantly (*p* < 0.01) identified in 14 chromosomes (A01, A02, A03, A04, A06, A10, B01, B03, B05, B06, B07, B08, B09, and B10). The QTLs with higher LOD scores were located on B06, A04, A02, A06, A03, A10, and B01. For the 134 genotyped BC_3_F_5_ plants, 602 loci scattered through 15 chromosomes were informative. In addition to A08 and B02, which lost the wild segments in BC_3_F_4_, all the loci on the chromosomes A05, A07, and B04 were homozygous for the cultivated alleles. Loci associated with LLS resistance were significantly (*p* < 0.01) identified in all the remaining chromosomes, except B05 (**Supplementary file 3**). The major QTLs, detected with higher LOD scores were located on A02, B06, A03, B01, A04, A10, and A06 (**Table 3**). A total of 257 BC_4_F_2_ plants, belonging to four families, were evaluated in the field for LLS and rust resistance, in 2019/2020, using a 1–9 grade scale. On the BC_4_F_2_ generation, only nine chromosomes (A02, A04, A06, A10, B01, B03, B06, B08, and B10) still had some wild alleles on their 449 informative loci. Loci linked to LLS resistance were significantly (*p* < 0.01) identified in six chromosomes, B06, A10, A04, B01, A06 and B10, in descending order of LOD scores. Therefore, the significant and major QTLs detected on all the four generations were located on chromosomes A04, A06, A10, B01, and B06. In addition, QTLs were identified on A02 with high LOD scores in all but the BC_4_F_2_ generation (**Table 3**). These six chromosome segments were considered as having the major QTLs for LLS resistance. The effects of cultivated x wild alleles of the nearest SNP of each identified QTL for LLS resistance on these six segments are shown in **Figure 4**. Differences were significant for all loci, according to a Kruskal–Wallis test (*p* < 0.01 and *p < 0.05), and show that the wild alleles significantly decrease LLS scores for all loci. Some BC_3_F_5_ plants containing the resistance chromosome segments from the wild species are shown in **Figure 5**, as compared to ‘IAC OL 3’. Complete QTL mapping information for the four populations is shown in **Supplementary file 3**.

**Table 3:** Quantitative trait loci identified for late leaf spot and rust resistance on populations of four generations (BC_3_F_2_, BC_3_F_4_, BC_3_F_5_ and BC_4_F_2_) derived from the cross ‘IAC OL 4’ × (*A. magna* K 30097 × *A. stenosperma* V 15076)^4x^ using the non-parametric interval mapping in R/qtl and a significance level of 1%, except the four QTLs with an asterisk (*) that were identified at 5%.

		BC_3_F_2_		BC_3_F_4_		BC_3_F_5_		BC_4_F_2_	
Chromosome	Segment (Mbp)	Position	LOD	Position	LOD	Position	LOD	Position	LOD
**Late leaf spot**									
**A02**	86 - 92	86.67	16.9	91.64	15.3	92.08	15.8	–	–
**A04**	0 - 7	6.68	5.4	0.11	19.4	3.02	9.4	3.71	4.3
**A06**	93 - 110	109.39	12.8	107.41	11.8	104.39	5.5	93.86*	3.2
**A10**	8 - 85	57.14	13.0	12.93	13.0	84.67	6.0	7.98	4.4
**B01**	0 - 2	1.43	7.7	1.13	11.2	1.43	9.6	2.10*	3.7
**B06**	130 - 134	129.58	14.9	133.74	22.8	129.58	13.9	133.74	6.6
**Rust**									
**B07**	114-116	114.49	7.0	114.49	4.3	115.51	3.7	–	–
**B08**	125 / 31	125.47	6.9	31.74*	3.6	31.74*	2.8	–	–

Chromosome segment and position, in Mbp, is based on BLAST similarity searches to the reference genomes of A. duranensis and A. ipaënsis (Bertioli et al., 2016). The symbol - means that no QTL was identified.

**Figure 6:**
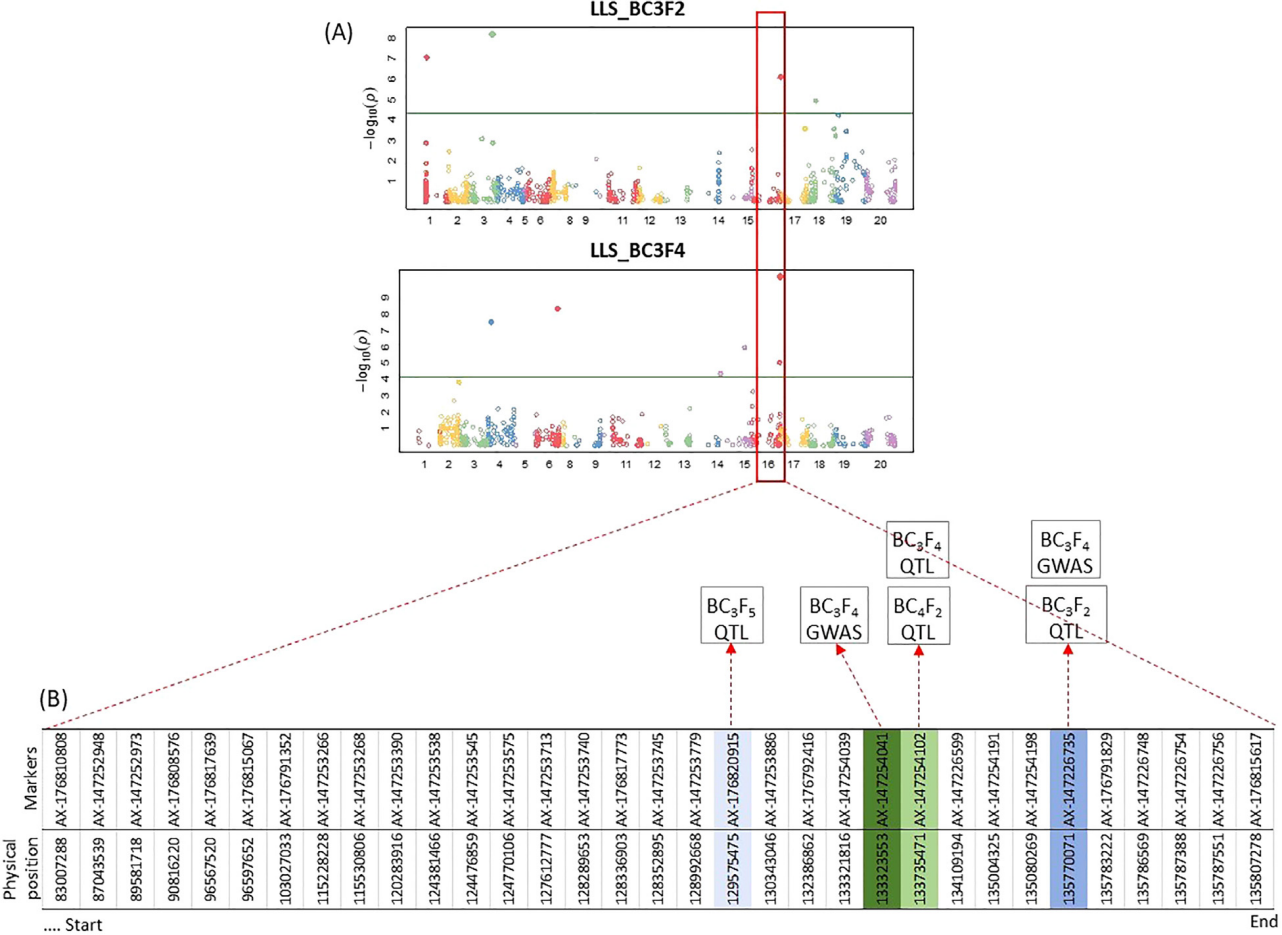
**(A)** Manhattan plot representing the results of GWAS of LLS using SNPs obtained from Axiom data for BC_3_F_2_ and BC_3_F_4_ generations. Each dot represents an SNP. The green line represents significance threshold (FDR = 0.05). No significant SNPs were identified for BC_3_F_5_ and BC_4_F_2_. **(B)** Distribution of significant markers identified from QTL and GWAS analyses in chromosome B06 on populations BC_3_F_2_, BC_3_F_4_, BC_3_F_5_, and BC_4_F_2_. Colored boxes of markers represent significant SNPs/QTLs from the GWAS/QTL analyses for the different generations.

GWAS analysis did not identify any significant marker on populations BC_3_F_5_ and BC_4_F_2_, but showed some significant SNPs on BC_3_F_2_ and BC_3_F_4_, especially on chromosome B06 (**Figure 6**). The significant markers identified by QTL mapping and GWAS analysis on the chromosome B06 are also shown in **Figure 6**.

For rust, QTLs were identified on 16 chromosomes in BC_3_F_2_ (except A03, A05, B01, and B04), 8 chromosomes in BC_3_F_4_ (A01, A04, A10, B04, B07, B08, B09, and B10), and 6 chromosomes in BC_3_F_5_ (A02, A03, A06, B03, B07, and B08) at a 5% significance level (**Supplementary file 3**). Only two common QTLs were detected, located on chromosomes B07 and B08 (**Table 3**). No QTLs were detected on BC_4_F_2_.

## Discussion

*Arachis hypogaea*, the cultivated peanut, has a very narrow genetic base and few sources of resistance to the major biotic and abiotic stresses that impair the crop productivity. This is especially true for the fungal foliar diseases, since no highly resistant genotype of pure cultivated peanut has been found to date. Crop wild relatives are the reservoir of many useful genes and highly resistant *Arachis* wild species and accessions have been identified (reviewed by Stalker, 2017). Despite this, the use of peanut relatives in crop improvement is still incipient and has been mainly hampered by the barriers of fertility due to the ploidy differences, the transfer of undesirable genes associated with the wild genes of interest (linkage drag), and the difficulties of monitoring the wild chromosomic segments being introgressed into the cultivars.

**Figure 7:**
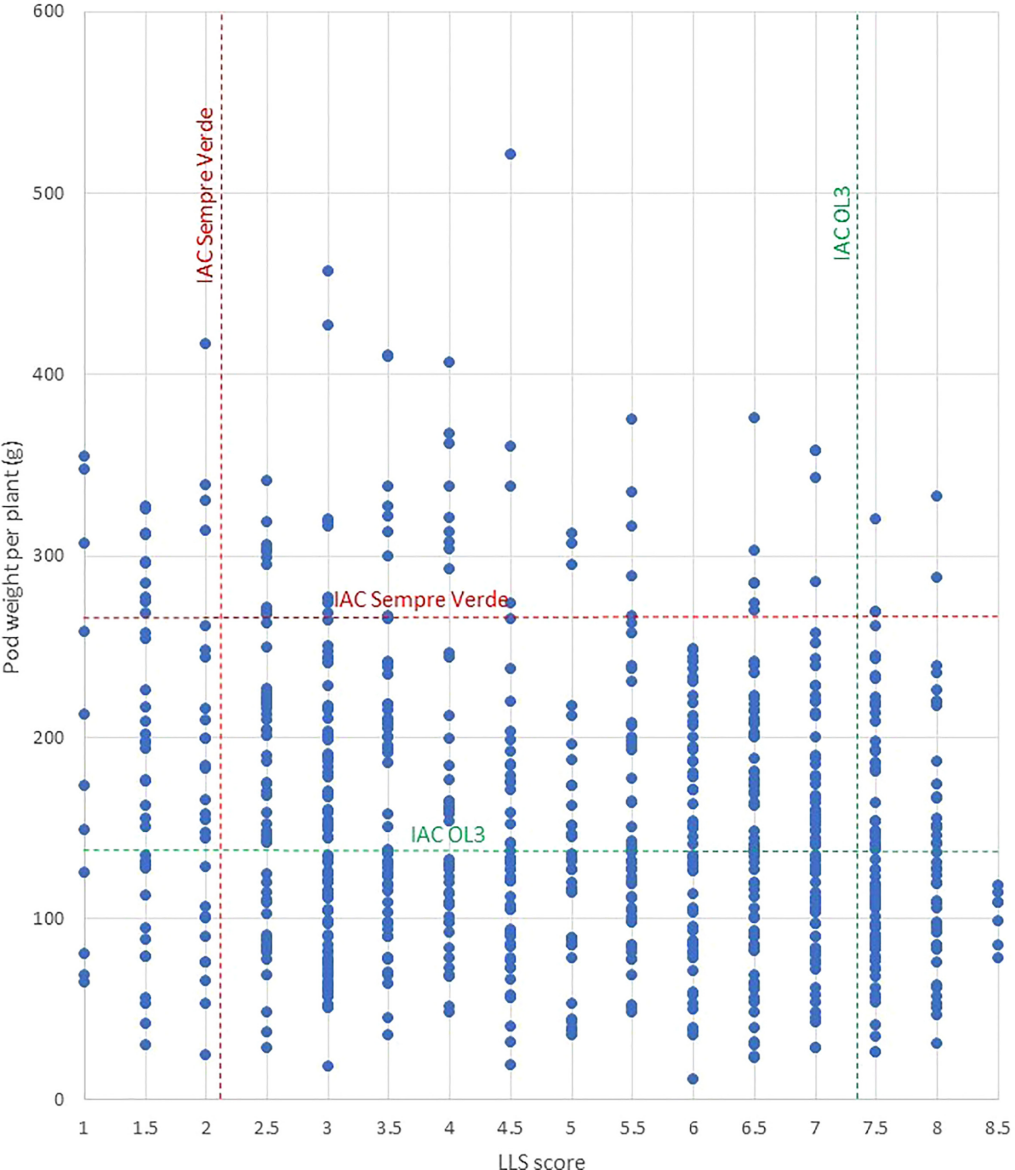
Pod weight produced and LLS score of each of the 808 plants of generations BC_3_F_2_, BC_3_F_3_, BC_3_F_5_, and BC_4_F_2_ and the controls ‘IAC Sempre Verde’ (red) and ‘IAC OL 3’ (green). No fungicide was sprayed on the four field assays.

To overcome the ploidy difference, we developed an induced allotetraploid by crossing the diploid B genome species *A. magna* accession K 30097 with the diploid A genome *A. stenosperma* accession V 15076, both highly resistant to LLS, early leaf spot, and rust (Fávero et al., 2015b; Leal-Bertioli et al., 2015a; Michelotto et al., 2015). The resulting allotetraploid also showed high resistance to LLS, early leaf spot, and rust (Michelotto et al., 2016). We used a backcross approach followed by selfings. For the efficient introgression of the genes of interest, we also identified and used microsatellite markers associated with resistance to rust in *A. magna* K 30097 (Leal-Bertioli et al., 2015a) and LLS in *A. stenosperma* V 10309 (Leal-Bertioli et al., 2009). Although this is not the same accession used in MagSten, we tentatively used and validated the markers on V 15076, which was found to be more resistant to LLS than V 10309 by Michelotto et al. (2015). Finally, for tracking the introgressed wild chromosome segments and speeding the recovery of the cultivated genome, we took advantage of the recently developed Thermofisher Axiom *Arachis* Arrays v.01 and v.02 (Clevenger et al., 2017a; Pandey et al., 2017a; Korani et al., 2019). These tools greatly reduced the barriers for the use of *Arachis* wild relatives in the peanut breeding program. We developed advanced lines containing wild chromosome segments conferring high resistance to two fungal foliar diseases, rust, and LLS. In addition, preliminary results suggest that some of them are also high-yielding lines (**Figure 7**).

Among these lines, 31 were highly resistant to LLS and productive under unsprayed fungicide conditions, and 5 of them showed resistance and production (considering the production of individual plants) similar to or higher than ‘IAC Sempre Verde’. This cultivar was released in Brazil in 2019 and incorporates very strong LLS and rust resistance (Godoy et al., 2022). Under cultivation without fungicide control of foliar diseases, it produces 5,000 kg ha^−1^. ‘IAC Sempre Verde’ provides the first viable option for ‘Organic’ production in the high disease pressure peanut growing environments in Brazil. During the development of ‘IAC Sempre Verde’, we discovered that its disease-resistant parent IAC 69007 has *A. cardenasii* chromosome segments that confer resistance to fungal foliar diseases located in chromosomes A02 and A03. Further investigations revealed similar segments in disease-resistant cultivars used in 30 countries in the Americas, Africa, Asia, and Oceania (Bertioli et al., 2021a). We consider it likely that *A. cardenasii* has been the main, and perhaps the only source of high resistance to fungal foliar diseases used in peanut breeding programs worldwide. Additional sources of resistance to these diseases are thus essential for the development of new peanut cultivars to reduce the risk of resistance breakdown. In this work, we identified resistances to LLS and rust provided by *A. stenosperma* and *A. magna* located mostly on different chromosomal locations from the *A. cardenasii* segments, providing new sources of resistance. Although QTLs were also detected on chromosome A02 of *A. stenosperma*, on the bottom end of the chromosome (from 86 to 92 Mbp, **Table 3**), they seem to be located in different segments from those of *A. cardenasii* that were located on the top of the chromosome (122,410 pb to 4.4 Mbp), using both the same physical map and SNP array (Lamon et al., 2020). In addition, some lines had a higher number of pods per plant than elite cultivars, indicating putative yield superiority. This will be tested in further field trials in large areas, cultivated under the recommended control of diseases for the crop. Some lines would also be tested as additional options for ‘Organic’ production, since this is a growing demand from the market in Brazil, and, more importantly, for conventional farming with reduced sprays. Utilization of these promising lines derived from wild *Arachis* species in peanut breeding programs will assist in developing new disease-resistant cultivars with a broader genetic base. Selected lines are also being crossed to highly resistant *A. cardenasii*-containing chromosome segment lines for pyramiding resistant genes, aiming at even greater resistance and durability.

QTL mapping, using the nonparametric interval mapping and 1,177 plants of BC_3_F_2_, BC_3_F_4_, BC_3_F_5_, and BC_4_F_2_, showed that six common segments had loci significantly associated with LLS resistance, located on chromosomes A02, A04, A06, A10, B01, and B06 (**Table 3**, **Figure 4**). These chromosome segments include the three segments containing the QTLs for LLS resistance previously identified in *A. stenosperma* V 10309, in chromosomes A02, A04, and A06 (Leal-Bertioli et al., 2009). These results corroborated and provided additional lines of evidence for the presence of major QTLs on these three chromosomes. Therefore, both accessions of *A. stenosperma* (V 10309 and V 15076) seem to share these LLS resistance genes. *Arachis stenosperma* has a peculiar disjunct distribution in Central Brazil and along the Atlantic coast, separated by more than 1,000 km. It is well known that *A. stenosperma* was cultivated by local people in the past, who probably promoted its migration to the coast (Krapovickas and Gregory, 1994; Custódio et al., 2005). The accession V 10309 was collected in the Mato Grosso state (Central Brazil), while V 15076 was collected in Paraná state, on the Atlantic coast. Studies have shown that all accessions of *A. stenosperma* studied to date are very closely related genetically (Singh et al., 2004; Koppolu et al., 2010; Moretzsohn et al., 2013) and morphologically similar (Krapovickas and Gregory, 1994). Based on this, we were confident that they should share some of the resistance segments, as corroborated by the present study.

Our QTL analyses also identified three additional chromosome segments associated with LLS resistance, on chromosomes A10, B01, and especially on B06, which showed QTLs with the highest or one of the highest LOD scores on all the four populations (**Table 3**). GWAS also identified significant markers on B06, very close to the mapped QTLs (**Figure 6**), which corroborated that the end of chromosome B06 (from 130 Mbp to 134 Mbp) on the induced allotetraploid MagSten has QTLs for resistance to LLS. Loci on A03, B07, and B09 were significantly associated on the initial generations with high LOD scores, but the wild alleles were lost on the following generations, since we were not marker-selecting segments on these chromosomes. BC_3_F_4_ or BC_3_F_5_ plants containing these segments will be rescued and advanced on our breeding schedule, for pyramiding of resistance QTLs for a more durable resistance. In addition, and as expected, several minor QTLs were detected in different chromosomes and populations (**Supplementary file 3**). The presence of some minor QTLs will also be monitored on the next cycles of selection.

Out of the 31 high-yielding lines, highly resistant to LLS and probably to rust, 23 have wild alleles on all the main six segments with QTLs conferring resistance to LLS, while the remaining eight lines have four or five of them (**Supplementary file 2**). In contrast, the most susceptible plants to LLS, considering the 1,177 genotyped and phenotyped plants of the four generations, have very few, if any, of the resistance segments. These results strongly suggest that the six chromosome segments are associated with LLS resistance, although the exact location of the resistance genes within the segments is still unknown. Additional evidence for the identified wild resistance segments was the clear decreasing of LLS scores on the successive BC_3_ generations, which were advanced by using a stringent foreground selection (**Figures 2**, **3**). The exception was BC_3_F_4_, which showed the higher minimum, average, and maximum disease values. In the 2018/2019 season, when the BC_3_F_4_ plants were evaluated, an early incidence of LLS occurred. Therefore, despite the plants being evaluated at approximately 120 days after sowing, as all the other generations, the disease damage was considerably higher than the other trials. Many ‘IAC OL 3’ plants were dead and LLS scores of 'IAC Sempre Verde' and ‘IAC Caiapó’ were higher than the values observed on the other seasons (**Table 1**).

Rust is a sporadic disease, and its incidence was very weak in the four BC_3_ field assays. Therefore, plants were evaluated by the presence (1) or absence (0) of rust lesions. In BC_4_F_2_, plants could be evaluated using the 1–9 grade scale. However, the incidence was only moderate and 228 out of 257 plants (88.7%) had scores lower than 2.0. Probably due to this low variation, no QTL was detected. On BC_3_, several QTLs were identified, but only two QTLs were significantly associated with all the three populations analyzed, using the nonparametric interval mapping and the argument model=“binary”, at *p* < 0.05 (**Table 3**). These QTLs were located on chromosomes B07 and B08. The segment on B07 was well defined, located within 114 and 116 Mbp, and the QTL with higher LOD scores on the three BC_3_ populations (**Table 3**; **Supplementary file 3**). The end of chromosome B08, where the QTL was identified on BC_3_F_2_ (at *p* < 0.01) coincides with the QTL previously identified for *A. magna* K 30097 on a diploid cross (Leal-Bertioli et al., 2015a). However, the QTLs were detected (at *p* < 0.05) close to 32 Mbp on BC_3_F_4_ and BC_3_F_5_. Therefore, the location of QTLs for rust resistance in *A. magna* still needs further investigation.

Very few QTLs for resistance to LLS or rust have been mapped in *Arachis* wild species. In contrast, a number of QTLs for resistance to both diseases have been mapped in cultivated peanut using *A. hypogaea* × *A. hypogaea* crosses, despite two out of the three sources of resistance used on these studies having wild species on their pedigrees. Using the *A. cardenasii-*derived GBPD4 as the source of resistance, the number of QTLs ranged from 4 to 28 for LLS resistance, and from 5 to 15 for rust resistance (Khedikar et al., 2010; Sujay et al., 2012; Kolekar et al., 2016). As expected, most of them are minor QTLs. However, two major and common QTL regions were detected, located on chromosomes A03 for LLS and rust resistance, and A02 for LLS resistance only. These two candidate genomic regions conferring resistance to LLS and rust have been validated and used on marker-assisted selection for developing foliar disease-resistant lines (Yeri and Bhat, 2016; Kolekar et al., 2017; Pandey et al., 2017b; Ahmad et al., 2020). It is now well established that both resistance regions came from *A. cardenasii* accession GKP 10017 (Bertioli et al., 2021a). QTL mapping studies using Tifrunner as the source of LLS resistance identified up to 22 QTLs, but three consistent QTLs, located on chromosomes A05, B03 and B05, were mapped and validated (Pandey et al., 2017c; Clevenger et al., 2018; Chu et al., 2019). Finally, a study using as the source of resistance the ICGV 86699 line, which is reported to be a derivative of *A. batizocoi/A. duranensis* × *A. hypogaea* “NC 2” crosses (Reddy et al., 1996), but which was found to have *A. cardenasii* segments from chromosomes A02 and A03 by Bertioli et al. (2021a), identified two major QTLs for LLS resistance on chromosomes B06 and A10 (Zhou et al., 2016). Therefore, from the six wild chromosome segments containing LLS resistance genes identified and introgressed into peanut here, only three were located on the same chromosomes of previously mapped QTLs: A02 from *A. cardenasii*, and A10 and B06, whose origin is unclear. At least the other three segments are new and have never been used by any breeding program. The co-localization or not of QTLs on chromosomes A02, A10, and B06 needs further investigation. For rust resistance, the present study provided additional lines of evidence for the presence of resistance genes on the chromosome B08. The two segments identified here (on chromosomes B07 and B08) do not coincide with the only other consistent QTL for rust resistance mapped to date, located on chromosome A03, from *A. cardenasii*.

**Figure 8:**
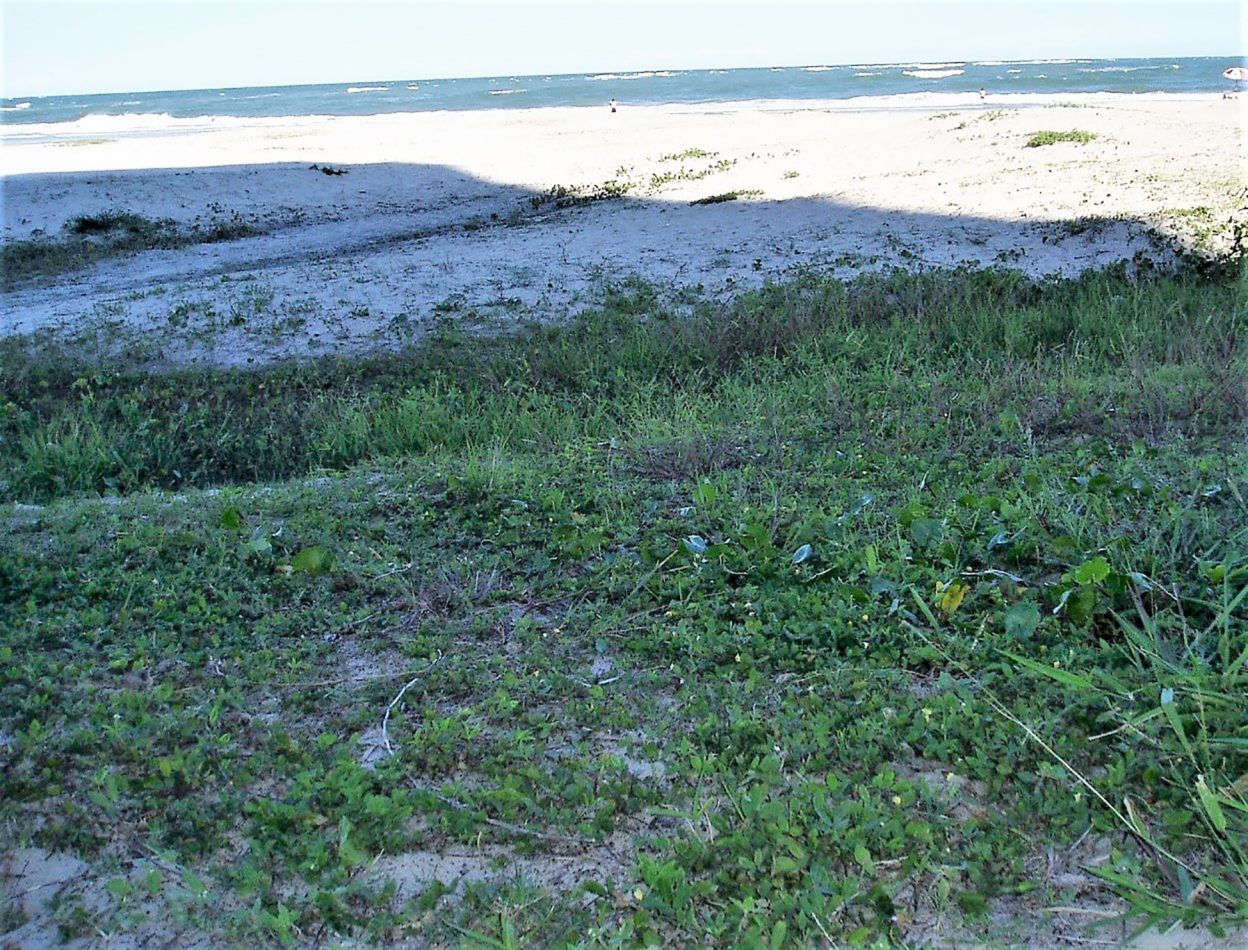
The accession *A. stenosperma* V 15076 growing in the place where it was collected, in Caiobá Beach, Paraná state. Note the pure sand where the plants were growing vigorously and the very close proximity to the sea. The photo was taken during its collection and kindly provided by the collector José Valls.

Here, we showed the efficient introgression of wild segments with resistance genes against two important diseases into an elite high oleic peanut cultivar from the secondary gene pool. The approach used in this study provides a way to expand the genetic base of cultivated peanut by exploiting genetic variability present in wild species, as well as ensuring a continuous supply of new sources of resistance to different biotic and abiotic stresses. As an example, the accession V 15076 of *A. stenosperma* was collected in the pure sand of a beach, very close to the sea, where it was found growing vigorously and producing many flowers (**Figure 8**) and seeds. This accession may also have tolerance to salinity, drought, and heat, among other stresses. Therefore, the material produced in the present study might also be important for the development of climate-resilient plants. Finally, we showed that genetic limits of a complex trait such as yield were also overcome during the introgression of segments from crop wild relatives.

## Data availability statement

The original contributions presented in the study are included in the article/**Supplementary Material**, further inquiries can be directed to the corresponding author.

## Author contributions

MCM, SCML-B, IJG, and DJB conceived the study. JFS, ARAM, MDM, and IJG conducted the field experiments. MCM, ARC, SCML-B, and DJB performed the molecular data analyses. MCM and JFS performed the QTL and statistical analyses. NM carried out GWAS analysis. The first draft of the manuscript was written by MCM, and all authors commented on previous versions of the manuscript. All authors contributed to the article and approved the submitted version.

## References

[B1] Ahmad S. Nawade B. Sangh C. Mishra G. P. Bosamia T. C. Radhakrishnan T.. (2020). Identification of novel QTLs for late leaf spot resistance and validation of a major rust QTL in peanut (*Arachis hypogaea* l.). 3 Biotech. 10, 1–13. doi: 10.1007/s13205-020-02446-4 33088655 PMC7527388

[B2] Backman P. A. Crawford M. A. (1984). Relationship between yield loss and severity of early and late leaf spot diseases of peanut (*Arachis hypogaea* l.). Phytopathology 74, 1101–1103. doi: 10.1094/Phyto-74-1101

[B3] Ballén-Taborda C. Chu Y. Ozias-Akins P. Timper P. Holbrook C. C. Jackson S. A.. (2019). A new source of root-knot nematode resistance from *Arachis stenosperma* incorporated into allotetraploid peanut (*Arachis hypogaea*). Sci. Rep. 9, 1–13. doi: 10.1038/s41598-019-54183-1 31776412 PMC6881346

[B4] Bertioli D. J. Cannon S. B. Froenicke L. Huang G. Farmer A. D. Cannon E. K.. (2016). The genome sequence of Arachis duranensis and Arachis ipaënsis, the diploid ancestors of cultivated peanut.). Nat. Genet. 48, 438–446. doi: 10.1038/ng.3517 26901068

[B5] Bertioli D. J. Clevenger J. Godoy I. J. Stalker H. T. Wood S. Santos J. F.. (2021a). Legacy genetics of *Arachis cardenasii* in the peanut crop shows the profound benefits of international seed exchange. Proc. Natl. Acad. Sci. U. S. A. 118, 1–9. doi: 10.1073/pnas.2104899118 PMC8463892 34518223

[B6] Bertioli D. J. Gao D. Ballen-Taborda C. Chu Y. Ozias-Akins P. Jackson S. A.. (2021b). Registration of GA-BatSten1 and GA-MagSten1, two induced allotetraploids derived from peanut wild relatives with superior resistance to leaf spots, rust, and root-knot nematode. J. Plant Regist. 15, 1–7. doi: 10.1002/plr2.20133

[B7] Bharadwaj C. Jorben J. Rao A. Roorkiwal M. Patil B. S. Jayalakshmi. (2022). Development of high yielding fusarium wilt resistant cultivar by pyramiding of "Genes" through marker-assisted backcrossing in chickpea (*Cicer arietinum* l.). Front. Genet. 13. doi: 10.3389/fgene.2022.924287 PMC9388742 35991541

[B8] Broman K. W. Wu H. Sen S. Churchill. G. A. (2003). R/qtl: QTL mapping in experimental crosses. Bioinform 19 (7), 889–890. doi: 10.1093/bioinformatics/btg112 12724300

[B9] Burow M. D. Simpson C. E. Starr J. L. Paterson A. H. (2001). Transmission genetics of chromatin from a synthetic amphidiploid to cultivated peanut (*Arachis hypogaea* l.): Broadening the gene pool of a monophyletic polyploid species. Genetics 159, 823–837. doi: 10.1093/genetics/159.2.823 11606556 PMC1461827

[B10] Chu Y. Chee P. Culbreath A. Isleib T. G. Holbrook C. C. Ozias-Akins P. (2019). Major QTLs for resistance to early and late leaf spot diseases are identified on chromosomes 3 and 5 in peanut (*Arachis hypogaea*). Front. Plant Sci. 10. doi: 10.3389/fpls.2019.00883 PMC6625158 31333711

[B11] Chu Y. Holbrook C. C. Timper P. Ozias-Akins P. (2007). Development of a PCR-based molecular marker to select for nematode resistance in peanut. Crop Sci. 47, 841–847. doi: 10.2135/cropsci2006.07.0474

[B12] Chu Y. Wu C. L. Holbrook C. C. Tillman B. L. Person G. Ozias-Akins P. (2011). Marker-assisted selection to pyramid nematode resistance and the high oleic trait in peanut. Plant Genome J. 4, 110. doi: 10.3835/plantgenome2011.01.0001

[B13] Clevenger J. Chu Y. Arrais Guimaraes L. Maia T. Bertioli D. Leal-Bertioli S.. (2017b). Gene expression profiling describes the genetic regulation of *Meloidogyne arenaria* resistance in *Arachis hypogaea* and reveals a candidate gene for resistance. Sci. Rep. 7, 1317. doi: 10.1038/s41598-017-00971-6 28465503 PMC5430994

[B14] Clevenger J. Chu Y. Chavarro C. Agarwal G. Bertioli D. J. Leal-Bertioli S. C. M.. (2017a). Genome-wide SNP genotyping resolves signatures of selection and tetrasomic recombination in peanut. Mol. Plant 10, 309–322. doi: 10.1016/j.molp.2016.11.015 27993622 PMC5315502

[B15] Clevenger J. Chu Y. Chavarro C. Botton S. Culbreath A. Isleib T. G.. (2018). Mapping late leaf spot resistance in peanut (*Arachis hypogaea*) using QTL-seq reveals markers for marker-assisted selection. Front. Plant Sci. 9. doi: 10.3389/fpls.2018.00083 PMC5807350 29459876

[B16] CONAB - Companhia Nacional de Abastecimento (2023). Available at: https://www.conab.gov.br/info-agro/safras/serie-historica-das-safras/itemlist/category/899-amendoim (Accessed 10 January 2023).

[B17] Coutinho W. M. Suassuna N. D. (2014). “Manejo integrado de doenças,” in Sistema de produção de amendoim, 2. ed. Ed. Suassuna T. M. F. (Campina Grande: Embrapa Algodão). Available at: https://www.spo.cnptia.embrapa.br/temas-publicados.

[B18] Custódio A. R. Peñaloza A. P. S. Valls J. F. M. (2005). Further cytogenetic information on *Arachis stenosperma* (Leguminosae). Cytologia (Tokyo). 70, 331–335. doi: 10.1508/cytologia.70.331

[B19] Custódio A. R. Seijo G. Valls J. F. M. (2013). Characterization of Brazilian accessions of wild *Arachis* species of section *Arachis* (Fabaceae) using heterochromatin detection and fluorescence *in situ* hybridization (FISH). Genet. Mol. Biol. 36, 364–370. doi: 10.1590/S1415-47572013000300011 24130444 PMC3795162

[B20] FAOSTAT – Food and Agriculture Organization of the United Nations (2022). Available at: https://www.fao.org/faostat/en/#data/QCL (Accessed 10 January 2023).

[B21] Fávero A. P. Pádua J. G. Costa T. S. Gimenes M. A. Godoy I. J. Moretzsohn M. C.. (2015b). New hybrids from peanut (*Arachis hypogaea* l.) and synthetic amphidiploid crosses show promise in increasing pest and disease tolerance. Genet. Mol. Res. 14, 16694–16703. doi: 10.4238/2015.December.11.17 26681016

[B22] Fávero A. P. Santos R. F. Simpson C. E. Valls J. F. M. Vello N. A. (2015a). Successful crosses between fungal-resistant wild species of *Arachis* (Section *Arachis*) and *Arachis hypogaea* . Genet. Mol. Biol. 38, 353–365. doi: 10.1590/S1415-475738320140376 26500440 PMC4612608

[B23] Fávero A. P. Simpson C. E. Valls J. F. M. Vello N. A. (2006). Study of the evolution of cultivated peanut through crossability studies among *Arachis ipaënsis, a. duranensis*, and *A. hypogaea* . Crop Sci. 46, 1546–1552. doi: 10.2135/cropsci2005.09-0331

[B24] Fernandez A. Krapovickas A. (1994). Cromosomas y evolución en *Arachis* (Leguminosae). Bonplandia 8, 187–220. doi: 10.30972/bon.81-41499

[B25] Frisch M. Melchinger A. E. (2005). Selection theory for marker-assisted back-crossing. Genetics 170, 909–917. doi: 10.1534/genetics.104.035451 15802512 PMC1450430

[B26] Gao D. Araujo A. C. G. Nascimento E. F. M. B. Chavarro M. C. Xia H. Jackson S. A.. (2021). ValSten: A new wild species derived allotetraploid for increasing genetic diversity of the peanut crop (*Arachis hypogaea* l.). Genet. Resour. Crop Evol. 68, 1471–1485. doi: 10.1007/s10722-020-01076-2

[B27] Godoy I. J. Santos J. F. Carvalho C. R. L. Michelotto M. D. Bolonhezi D. Freitas R. S.. (2014). IAC OL 3 and IAC OL 4: New Brazilian peanut cultivars with the high oleic trait. Crop Breed. Appl. Biotechnol. 14, 200–203. doi: 10.1590/1984-70332014v14n3a30

[B28] Godoy I. J. Santos J. F. Moretzsohn M. C. Moraes A. R. A. Michelotto M. D. Bolonhezi D.. (2022). ‘IAC SEMPRE VERDE’: A wild-derived peanut cultivar highly resistant to foliar diseases. Crop Breed. Appl. Biotechnol. 22, e41252232. doi: 10.1590/1984-70332022v22n3c25

[B29] Holbrook C. C. Ozias-Akins P. Chu Y. Lamon S. Bertioli D. J. Leal-Bertioli S. C.. (2022). Registration of TifGP-3 and TifGP-4 peanut germplasm lines. J. Plant Regist. 16, 120–123. doi: 10.1002/plr2.20179

[B30] Khedikar Y. P. Gowda M. V. C. Sarvamangala C. Patgar K. V. Upadhyaya H. D. Varshney R. A. (2010). QTL study on late leaf spot and rust revealed one major QTL for molecular breeding for rust resistance in groundnut (*Arachis hypogaea* l.). K Theor. Appl. Genet. 121, 971–984. doi: 10.1007/s00122-010-1366-x PMC2921499 20526757

[B31] Kim M. S. Yu J. K. Ko S. R. Kim K. J. Ji H. Kang K. K.. (2022). Marker-assisted backcrossing (MABc) to improve eating quality with thin seed coat and aleurone layer of non-glutinous japonica variety in rice. Genes 13, 210. doi: 10.3390/genes13020210 35205255 PMC8872511

[B32] Kolekar R. M. Sujay V. Shirasawa K. Sukruth M. Khedikar Y. P. Gowda M. V. C.. (2016). QTL mapping for late leaf spot and rust resistance using an improved genetic map and extensive phenotypic data on a recombinant inbred line population in peanut (*Arachis hypogaea* l.). Euphytica 209, 147–156. doi: 10.1007/s10681-016-1651-0

[B33] Kolekar R. M. Sukruth M. Shirasawa K. Nadaf H. L. Motagi B. N. Lingaraju S.. (2017). Marker-assisted backcrossing to develop foliar disease-resistant genotypes in TMV 2 variety of peanut (*Arachis hypogaea* l.). Plant Breed. 136, 948–953. doi: 10.1111/pbr.12549

[B34] Koppolu R. Upadhyaya H. D. Dwivedi S. L. Hoisington D. A. Varshney R. K. (2010). Genetic relationships among seven sections of genus *Arachis* studied by using SSR markers. BMC Plant Biol. 10, 15. doi: 10.1186/1471-2229-10-15 20089171 PMC2826335

[B35] Korani W. Clevenger J. P. Chu Y. Ozias-Akins P. (2019). Machine learning as an effective method for identifying true single nucleotide polymorphisms in polyploid plants. Plant Genome 12, 1–10. doi: 10.3835/plantgenome2018.05.0023 PMC12962348 30951095

[B36] Krapovickas A. Gregory W. C. (1994). Taxonomia del genero *Arachis* (Leguminosae). Bonplandia 8, 1–186.

[B37] Kumari V. Gowda M. V. C. Tasiwal V. Pandey M. K. Bhat R. S. Mallikarjuna N.. (2014). Diversification of primary gene pool through introgression of resistance to foliar diseases from synthetic amphidiploids to cultivated groundnut (*Arachis hypogaea* l.). Crop J. 2, 110–119. doi: 10.1016/j.cj.2014.03.002

[B38] Lamon S. Chu Y. Guimaraes L. A. Bertioli D. J. Leal-Bertioli S. C. M. Santos J. F.. (2020). Characterization of peanut lines with interspecific introgressions conferring late leaf spot resistance. Crop Sci. 61, 1724–1738. doi: 10.1002/csc2.20414

[B39] Lavia G. I. Ortiz A. M. Fernández A. (2009). Karyotypic studies in wild germplasm of *Arachis* (Leguminosae). Genet. Resour. Crop Evol. 56, 755–764. doi: 10.1007/s10722-008-9399-6

[B40] Leal-Bertioli S. C. M. Cavalcante U. Gouvea E. G. Ballén-Taborda C. Shirasawa K. Guimarães P. M.. (2015a). Identification of QTLs for rust resistance in the peanut wild species *Arachis magna* and the development of KASP markers for marker-assisted selection. G3; Genes|Genomes|Genetics 5, 1403–1413. doi: 10.1534/g3.115.018796 25943521 PMC4502374

[B41] Leal-Bertioli S. C. M. José A. C. V. F. Alves-Freitas D. M. T. Moretzsohn M. C. Guimarães P. M. Nielen S.. (2009). Identification of candidate genome regions controlling disease resistance in *Arachis* . BMC Plant Biol. 9, 112. doi: 10.1186/1471-2229-9-112 19698131 PMC2739205

[B42] Leal-Bertioli S. C. M. Moretzsohn M. C. Roberts P. A. Ballén-Taborda C. Borba T. C. O. Valdisser P. A.. (2016). Genetic mapping of resistance to *Meloidogyne arenaria* in *Arachis stenosperma*: A new source of nematode resistance for peanut. G3; Genes|Genomes|Genetics 6, 377–390. doi: 10.1534/g3.115.023044 PMC4751557 26656152

[B43] Leal-Bertioli S. C. M. Moretzsohn M. C. Santos S. P. Brasileiro A. C. M. Guimarães P. M. Bertioli D. J.. (2017). Phenotypic effects of allotetraploidization of wild *Arachis* and their implications for peanut domestication. Am. J. Bot. 104, 379–388. doi: 10.3732/ajb.1600402 28341626

[B44] Leal-Bertioli S. C. M. Santos S. P. Dantas K. M. Inglis P. W. Nielen S. Araujo A. C. G.. (2015b). *Arachis batizocoi*: A study of its relationship to cultivated peanut (*A. hypogaea*) and its potential for introgression of wild genes into the peanut crop using induced allotetraploids. Ann. Bot. 115, 237–249. doi: 10.1093/aob/mcu237 25538110 PMC4551086

[B45] Mendiburu F. G. Agricolae: Statistical Procedures for Agricultural Research. R package version 1.3.5. Available at: https://CRAN.R-project.org/package=agricolae.

[B46] Michelotto M. D. Barioni W. Resende M. D. V. Godoy I. J. Leonardecz E. Fávero A. P. (2015). Identification of fungus resistant wild accessions and interspecific hybrids of the genus *Arachis* . PloS One 10, 1–17. doi: 10.1371/journal.pone.0128811 PMC4474867 26090811

[B47] Michelotto M. D. Godoy I. J. Santos J. F. Martins A. L. M. Leonardecz E. Fávero A. P. (2016). Identifying *Arachis* amphidiploids resistant to foliar fungal diseases. Crop Sci. 56, 1792–1798. doi: 10.2135/cropsci2015.06.0393

[B48] Moraes A. S. Godoy I. J. (1997). “Amendoim - controle de doenças,” in Controle de doenças de plantas: Grandes culturas, vol. 1 . Eds. Zambolim L. Vale F. X. R. (Viçosa, Universidade Federal de Viçosa; Brasília, Ministério da Agricultura e do Abastecimento), 1–49.

[B49] Moretzsohn M. C. Gouvea E. G. Inglis P. W. Leal-Bertioli S. C. M. Valls J. F. M. Bertioli D. J. (2013). A study of the relationships of cultivated peanut (*Arachis hypogaea*) and its most closely related wild species using intron sequences and microsatellite markers. Ann. Bot. 111, 113–126. doi: 10.1093/aob/mcs237 23131301 PMC3523650

[B50] Pandey M. K. Agarwal G. Kale S. M. Clevenger J. Nayak S. N. Sriswathi M.. (2017a). Development and evaluation of a high density genotyping “Axiom-*Arachis*” array with 58 K SNPs for accelerating genetics and breeding in groundnut. Sci. Rep. 7, 1–10. doi: 10.1038/srep40577 28091575 PMC5238394

[B51] Pandey M. K. Khan A. W. Singh V. K. Vishwakarma M. K. Shasidhar Y. Kumar V.. (2017b). QTL-seq approach identified genomic regions and diagnostic markers for rust and late leaf spot resistance in groundnut (*Arachis hypogaea* l.). Plant Biotechnol. J. 15, 927–941. doi: 10.1111/pbi.12686 28028892 PMC5506652

[B52] Pandey M. K. Wang H. Khera P. Vishwakarma M. K. Kale S. M. Culbreath A. K.. (2017c). Genetic dissection of novel QTLs for resistance to leaf spots and tomato spotted wilt virus in peanut (*Arachis hypogaea* l.). Front. Plant Sci. 8. doi: 10.3389/fpls.2017.00025 PMC5281592 28197153

[B53] Reddy L. J. Nigam S. N. Moss J. P. Singh A. K. Subrahmanyam P. McDonald D.. (1996). Registration of ICGV 86699 peanut germplasm line with multiple disease and insect resistance. Crop Sci. 36, 821. doi: 10.2135/cropsci1996.0011183X003600030072x

[B54] Robledo G. Seijo G. (2010). Species relationships among the wild b genome of *Arachis* species (section *Arachis*) based on FISH mapping of rDNA loci and heterochromatin detection: A new proposal for genome arrangement. Theor. Appl. Genet. 121, 1033–1046. doi: 10.1007/s00122-010-1369-7 20552326

[B55] Seijo G. J. Atahuachi M. Simpson C. E. Krapovickas A. (2021). *Arachis inflata*: A new b genome species of *Arachis* (Fabaceae). Bonplandia 30, 1–6. doi: 10.30972/bon.3024942

[B56] Shasidhar Y. Variath M. T. Vishwakarma M. K. Manohar S. S. Gangurde S. S. Sriswathi M.. (2020). Improvement of three popular Indian groundnut varieties for foliar disease resistance and high oleic acid using SSR markers and SNP array in marker-assisted backcrossing. Crop J. 8, 1–15. doi: 10.1016/J.CJ.2019.07.001

[B57] Shokes F. M. Culbreath A. K. (1997). “Early and late leaf spots,” in Compendium of peanut diseases, 2nd ed. Eds. Kokalis-Burelle N. Porter D. M. Rodríguez-Kábana R. Smith D. H. Subrahmanyam P. (St Paul, MN, USA: The American Phytopathological Society). APS Press.

[B58] Silvestri M. C. Ortiz A. M. Lavia G. I. (2015). rDNA loci and heterochromatin positions support a distinct genome type for “x = 9 species” of section *Arachis* (*Arachis*, leguminosae). Plant Syst. Evol. 301, 555–562. doi: 10.1007/s00606-014-1092-y

[B59] Simpson C. E. (1991). Pathways for introgression of pest resistance into *Arachis hypogaea* l. Peanut Sci. 18, 22–26. doi: 10.3146/i0095-3679-18-1-8

[B60] Simpson C. E. Krapovickas A. Valls J. F. M. (2001). History of *Arachis* including evidence of *A. hypogaea* l. progenitors. Peanut Sci. 28, 78–80. doi: 10.3146/i0095-3679-28-2-7

[B61] Simpson C. E. Starr J. L. Baring M. R. Burow M. D. Cason J. M. Wilson J. N. (2013). Registration of ‘Webb’ peanut. J. Plant Regist. 7, 265–268. doi: 10.3198/jpr2013.01.0005crc

[B62] Singh M. P. Erickson J. E. Boote K. J. Tillman B. J. Jones J. W. van Bruggen A. H. C. (2011). Late leaf spot effects on growth, photosynthesis, and yield in peanut cultivars of differing resistance. Agron. J. 103, 85–91. doi: 10.2134/agronj2010.0322

[B63] Singh A. K. Smartt J. Singh R. (2004). Variation studies in a wild groundnut species, *Arachis stenosperma* krapov. & W.C. Gregory nov. sp. Plant Genet. Resour. 2, 99–106. doi: 10.1079/PGR200437

[B64] Smartt J. Gregory W. C. Gregory M. P. (1978). The genomes of *Arachis hypogaea.* 1. cytogenetic studies of putative genome donors. Euphytica 27, 665–675. doi: 10.1007/BF00023701

[B65] Stalker H. T. (1991). A new species in section *Arachis* of peanuts with a d genome. Am. J. Bot. 78, 630–637. doi: 10.1002/j.1537-2197.1991.tb12587.x

[B66] Stalker H. T. (2017). Utilizing wild species for peanut improvement. Crop Sci. 57, 1102–1120. doi: 10.2135/cropsci2016.09.0824

[B67] Subrahmanyam P. Mcdonald D. Waliyar F. Reddy L. J. Nigam S. N. Gibbons R. W.. (1995). Screening methods and sources of resistance to rust and late leaf spot of groundnut. Inf. Bull. 47, 1–26. Patancheru: ICRISAT.

[B68] Sujay V. Gowda M. V. C. Pandey M. K. Bhat R. S. Khedikar Y. P. Nadaf H. L.. (2012). Quantitative trait locus analysis and construction of consensus genetic map for foliar disease resistance based on two recombinant inbred line populations in cultivated groundnut (*Arachis hypogaea* l.). Mol. Breed. 30, 773–788. doi: 10.1007/s11032-011-9661-z 22924018 PMC3410029

[B69] Valls J. F. M. Costa L. C. Custódio A. R. (2013). A novel trifoliolate species of *Arachis* (Fabaceae) and further comments on taxonomic section trierectoides. Bonplandia 22, 91–97. doi: 10.30972/bon.2211257

[B70] Valls J. F. M. Simpson C. E. (2005). New species of *Arachis* (Leguminosae) from Brazil, Paraguay and Bolivia. Bonplandia 14, 35–63. doi: 10.30972/bon.141-21387

[B71] Valls J. F. M. Simpson C. E. (2017). A new species of *Arachis* (Fabaceae) from mato grosso, Brazil, related to *Arachis matiensis* . Bonplandia 26, 143–149. doi: 10.30972/bon.2622575

[B72] Varshney R. K. Pandey M. K. Janila P. Nigam S. N. Sudini H. Gowda M. V. C.. (2014). Marker−assisted introgression of a QTL region to improve rust resistance in three elite and popular varieties of peanut (*Arachis hypogaea* l.). Theor. Appl. Genet. 127, 1771–1781. doi: 10.1007/s00122-014-2338-3 24927821 PMC4110420

[B73] Wang S. Basten C. J. Zeng Z. B. (2006). Windows QTL cartographer 2.5. department of statistics (Raleigh, NC: North Carolina State University).

[B74] Yeri S. B. Bhat R. S. (2016). Development of late leaf spot and rust resistant backcross lines in jl 24 variety of groundnut (*Arachis hypogaea* l.). Electron. J. Plant Breed. 7, 37–41. doi: 10.5958/0975-928X.2016.00005.3

[B75] Zhou X. Xia Y. Liao J. Liu K. Li Q. Dong Y.. (2016). Quantitative trait locus analysis of late leaf spot resistance and plant-type-related traits in cultivated peanut (*Arachis hypogaea* l.) under multi-environments. PloS One 11, 1–18. doi: 10.1371/journal.pone.0166873 PMC5117734 27870916

